# Influence of Thoracic Trauma on Fracture Healing in Long Bones—A Retrospective Analysis

**DOI:** 10.3390/jcm11030717

**Published:** 2022-01-28

**Authors:** Karsten Timm, Nike Walter, Martin Heinrich, Gero Knapp, Ulrich Thormann, Thaqif El Khassawna, Volker Alt, Christian Heiss, Markus Rupp

**Affiliations:** 1 Department of Trauma, Hand and Reconstructive Surgery, University Hospital Giessen GmbH, 35392 Giessen, Germany; karsten.timm@med.uni-giessen.de (K.T.); martin.heinrich@chiru.med.uni-giessen.de (M.H.); gero.knapp@chiru.med.uni-giessen.de (G.K.); ulrich.thormann@chiru.med.uni-giessen.de (U.T.); thaqif.elkhassawna@chiru.med.uni-giessen.de (T.E.K.); office.uch@ukr.de (V.A.); christian.heiss@chiru.med.uni-giessen.de (C.H.); 2 Department of Trauma Surgery, University Hospital Regensburg, 93053 Regensburg, Germany; nike.walter@ukr.de; 3 Laboratory for Experimental Trauma Surgery, Justus-Liebig-University Giessen, 35392 Giessen, Germany

**Keywords:** fracture healing, chest trauma, thoracic trauma, bone consolidation, nonunion, RUST

## Abstract

Purpose: Pre-clinical studies indicate that concomitant thoracic trauma impairs fracture healing of long bones and reduces callus formation. The aim of this study was to investigate whether patients with accompanying chest trauma suffer from delayed fracture healing of long bones in comparison with patients with fractures of two long bones or isolated fractures. Patients and Methods: This is a clinical retrospective study from a level I trauma center. The patients were divided into three groups: (1) thoracic trauma and fracture of a long bone, (2) fractures of two long bones, (3) isolated fracture of a long bone. The fracture consolidation was defined using the radiographic union scale in tibial fractures (RUST). A RUST value of ≥10 six-to-eight months after definitive operative intervention represented complete fracture healing. Results: In the first group 19 (43.2%) fractures did not show full consolidation, in the second group 14 (45.2%) and 13 (41.9%) and in the third group 14 (36.8%). The analysis revealed no statistically significant differences between the groups regarding consolidation of the fractures six-to-eight months after definitive operative intervention (*p* = 0.84). Conclusions: Unlike previously reported pre-clinical data, this study did not demonstrate a negative effect on fracture consolidation in long bones when accompanied by thoracic trauma. Furthermore, the results demonstrated that concomitant fractures of two long bones does not have a negative effect on fracture consolidation.

## 1. Introduction

Despite enormous efforts in research, monetary investments and extensive observation in everyday clinical practice, bone healing is still not fully understood. Fracture healing is a very complex process that depends on many different factors [1,2,3,4]. Besides bone metabolism, bone healing is influenced by trauma mechanisms, accompanying soft tissue injury, treatment strategy and comorbidities [1,2,4,5]. Furthermore, injuries to other organs influence fracture healing via the release of inflammatory mediators such as cytokines [5]. For instance, accelerated fracture healing due to concomitant traumatic brain injury (TBI) has been demonstrated in clinical and pre-clinical studies [6,7,8]. Those data suggested a systemic interplay in multiple injuries leading to either an adverse or a beneficial effect on long bone fracture healing. Up to date, no clinical data supported the pre-clinical reports of impaired fracture healing and reduced callus formation in long bones when accompanied by thoracic trauma. In rat models, reduced callus volume and biomechanical stability has been demonstrated in fractures accompanied by chest trauma [9,10]. Additional soft tissue trauma further impaired fracture healing [9,11,12]. Intriguingly, Recknagel et al. revealed that a chest trauma triggers a post-traumatic systemic inflammation and thus, changes the callus formation and composition [13]. Differences in pulmonary oxygenation and application of an antagonist against the C5a receptor, which plays a crucial role in the complement cascade, were shown to reduce the diminishing effect of thoracic trauma on fracture healing [14,15].

Despite this pre-clinical data on chest trauma impairing long bone healing, no clinical studies have demonstrated the influence of chest trauma on fracture consolidation. The present study aimed to evaluate whether patients with fractures of long bones and an accompanying thoracic trauma have reduced fracture consolidation in comparison with patients with fractures of two long bones or patients with isolated long bone fractures. The study also aimed to assess the predictive value of thoracic trauma scores for fracture consolidation.

## 2. Patients and Methods

This study was approved by the institutional review board (IRB) of the Justus-Liebig-University Giessen “Ethics Committee of the Justus-Liebig-University Giessen” (16 April 2018, AZ 67/18). Informed consent was not required for this retrospective analysis as IRB approval was received. Patient data protection was ensured.

The records of 762 patients with long bone fractures treated between January 2008 and December 2017 were examined from the clinical database of the Department of Trauma, Hand and Reconstructive Surgery, University Hospital Giessen GmbH, Rudolf-Buchheim-Str. 7, 35392 Giessen, Germany. From these 762 patients a total of 113 patients were included in this study, divided into three groups. The inclusion criteria for all three groups were age ≥ 18 years and available X-ray images of the fracture six-to-eight months after definitive operative intervention. For the first group, a thoracic CT or thoracic X-ray had to be available upon admission. Exclusion criteria for all three groups were being aged < 18 years, a lethal hospital stay of the patient or a moderate or severe traumatic brain injury (Glasgow Coma Scale (GCS) 3–12). However, patients with other accompanying injuries such as facial, abdominal, soft tissue injuries or spinal injuries without neurological symptoms were not excluded.

The first group (control group) included 44 patients who suffered from a thoracic trauma and a fracture of a long bone [TXT+FX]. The second group (comparison group) comprised 31 patients who suffered from fractures of two long bones [FX+FX]. These fractures were randomly divided into two subgroups (fracture 1 and fracture 2). The third group (further comparison group) had 38 patients who suffered from an isolated fracture of a long bone [FX]. We retrospectively compared the data between the three groups. In a retrospective assessment of the medical records the following variables were recorded: age, sex, body mass index (BMI), comorbidities, trauma mechanism, length of hospital stay, anatomical fracture localization, fracture management, distribution of thoracic injuries, American Society of Anesthesiologists (ASA) classification [16], injury severity score (ISS) [17] and GCS [18] at hospital admittance. Thoracic trauma severity was assessed using the pulmonary contusion score (PCS) [19], the thoracic trauma severity score (TTS) [19,20] and the cumulated abbreviated injury scale chest (AIS_Chest_) [19,21]. Since the oxygenation index of the patients was not documented or visible in the present data set, the minimum (TTS_min._) and maximum (TTS_max._) scores of the Horowitz quotient were used to determine the TTS. This results in an approximate total point range in which the real TTS lies with a very high probability. Furthermore, fracture healing was assessed using the radiographic union scale in tibial fractures (RUST). The RUST is a radiological scoring system assessing fracture healing or rather callus formation in a standardized manner on conventional X-ray images [22,23]. To assess the healing process X-rays of the fractures were examined two-to-four weeks (point in time t_1_), five-to-seven weeks (point in time t_2_), eleven-to-13 weeks (point in time t_3_) and six-to-eight months (point in time t_4_) after the definitive operation. Definitive fracture consolidation was evaluated using the RUST at t_4_. Thus, a RUST ≥ 10 was rated as a consolidated fracture. A RUST < 10 was defined as unhealed fracture [22,23]. The assessment on the RUST was performed by one of the authors (K.T.).

### Statistical Analysis

Data were analyzed using Microsoft Excel Version 1809 (Microsoft Corporation, Microsoft Excel, Richmond, WA, USA) and SPSS statistics version 24.0 (IBM, SPSS Inc., Armonk, NY, USA, https://www.ibm.com, accessed on 10 October 2021). Descriptive statistics were calculated for all variables. Continuous variables were expressed as the mean and standard deviation. To determine that the data distribution was appropriate for parametric testing, Shapiro–Wilk and Levene’s tests were applied for each analysis, respectively. For comparisons between continuous variables the univariate ANOVA with a Tukey post-hoc test was performed. Regarding the comparison auf trauma scores between the groups, normal distribution and homogeneity of variances could not be assumed (Levene’s Test *p* < 0.05, Shapiro–Wilk test *p* < 0.05). Therefore, the Kruskal–Wallis-Test was used for comparison. Th chi-square test was used for comparison of categorical variables. Correlations were calculated using Pearson’s correlation coefficient r. The significance level was set at *p* < 0.05.

To calculate sample size G*power software 3. 1.9.7 (Heinrich Heine University, Dusseldorf, Germany) used [24]. The effect size was computed using the non-parametric effect size calculation method [25] and was determined to 0.5, the significance cutoff was set to a *p* value of 0.05 and the analysis power of 0.80. Taking three different groups into account with ten variables, a total sample size of 74 patients divided equally on three groups was calculated. Number of variables was determined to cover at least subjects’ demographic data and trauma scores (Table 1 and Table 2).

## 3. Results

A total of 762 patients were eligible for the study, out of which 113 patients met the inclusion criteria. Based on the combination of fractures patients were divided into the three groups: 44 in the [TXT+FX] group, 31 in the [FX+FX] group and 38 in the [FX] group. There were no statistically significant differences between the three groups regarding age (*p* = 0.22), gender (*p* = 0.28) or BMI (*p* = 0.72) (Table 1). With regard to the ISS, there were significant differences between [TXT+FX] and [FX+FX] (*p* < 0.001). Furthermore, the ISS between [TXT+FX] and [FX] (*p* < 0.001) differed significantly, but not between [FX+FX] and [FX] (*p* = 0.66). The ASA-classification (H = 1.378, *p* = 0.50) and the GCS (H = 2.61, *p* = 0.27) did not differ between the three groups (Table 2).

### 3.1. Comorbidities and Medications

Table 3 presents the comorbidities of the examined patients. The secondary diagnoses showed no significant influence on the rate of fracture consolidation (r = 0.03, *p* = 0.75). Medications used during the stay at hospital were not analyzed.

### 3.2. Trauma Mechanism and Length of Hospital Stay

The trauma mechanism was differentiated into high -energy trauma/fall from height ≥ 3 m and low-energy trauma/fall from height < 3 m. In the [TXT+FX] group 37 (84.1%) patients, in the [FX+FX] group 20 (64.5%) patients and in the [FX] group 23 (60.5%) patients suffered from a high-energy trauma/fall from height ≥ 3 m. A low-energy trauma/fall from height < 3 m occurred in seven (15.9%) subjects in the [TXT+FX] group, eleven (35.5%) in the [FX+FX] group and 15 (39.5%) in the [FX] group (Table 4). The total length of hospital stay was 21.6 ± 12.1 days for [TXT+FX] group, 16.8 ± 7.9 days for [FX+FX] group and 14.4 ± 11.1 days for [FX] group (Table 4). There were no statistically significant differences between the groups regarding trauma mechanism (*p* = 0.04). However, the Tukey post-hoc test showed a significant difference (*p* = 0.009) between [TXT+FX] group and [FX] group regarding the total length of hospital stay (7.87; 99%-CI (0.08; 14.38)).

### 3.3. Fracture Management

Fracture treatment was divided into single-stage management and multi-stage management. Treatment options were operative with plate, nail, external fixator, screws or cerclage and k-wire and conservative (Table 5 and Table 6). In the [TXT+FX] group 29 (65.9%) patients underwent single-stage and 15 (34.1%) multi-stage management. In the [FX+FX] fracture 1 group 19 (61.3%) patients had single-stage and 12 (38.7%) patients had multi-stage treatment. In the [FX+FX] fracture 2 group 18 (58.1%) patients got single-stage and 13 (41.9%) multi-stage management. In the [FX] group 28 (73.7%) patients underwent single-stage and ten (26.3%) multi-stage treatment. There were no statistical differences between the three groups regarding fracture management (*p* = 0.54) using univariate ANOVA.

### 3.4. Thoracic Trauma

Thoracic trauma was differentiated into hemothorax, pneumothorax, pleural effusion, rib fracture (single or multiple), cardiac contusion, sternal fracture, lung contusion, chest contusion and lung laceration. Table 7 shows the distribution of thoracic injuries. The analyzed patients had either one thoracic trauma or a combination of thoracic injuries. Twenty-three patients presented with a single thoracic injury, nine patients showed two thoracic injuries, six patients had three thoracic injuries, three patients suffered from four thoracic injuries and five, six and eight thoracic injuries were present in one patient. There was no patient having seven or a combination of all thoracic injuries. There was no influence of the number of thoracic injuries on the consolidation of fractures (χ^2^(6) = 7.785, *p* = 0.249, φ = 0.42).

### 3.5. Fracture Consolidation

**Figure 1:**
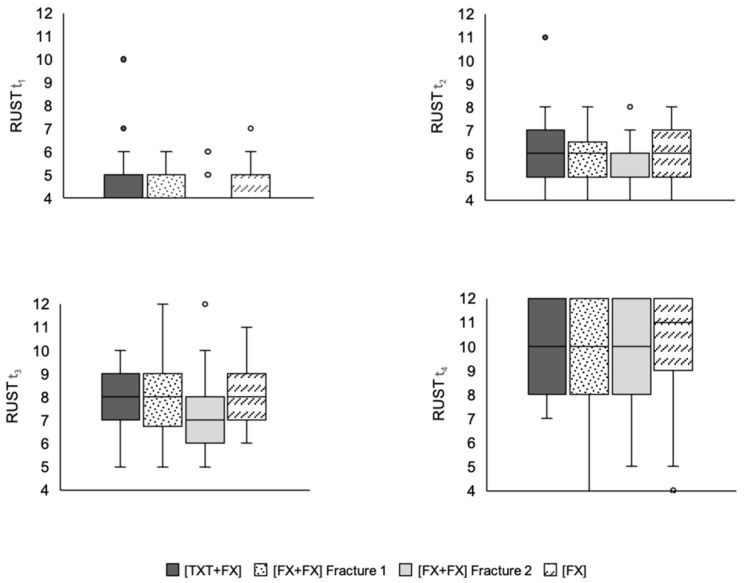
RUST at t_1_ to t_4_. The diagram shows the box plots of the RUST of the three examined groups at times t_1_–t_4_. The boxes contain the median as a horizontal line and are delimited by the upper and lower quartile. The range covers the entire scope of the RUST determined in each case. The points reflect statistical outliers. There were no statistically significant differences between the groups with regard to the RUST at time t_1_ (*p* = 0.09), t_2_ (*p* = 0.07), t_3_ (*p* = 0.28) and t_4_ (*p* = 0.49). [TXT+FX]—thoracic trauma and fracture of a long bone, [FX+FX]—fractures of two long bones, [FX]—isolated fracture of a long bone.

**Figure 2:**
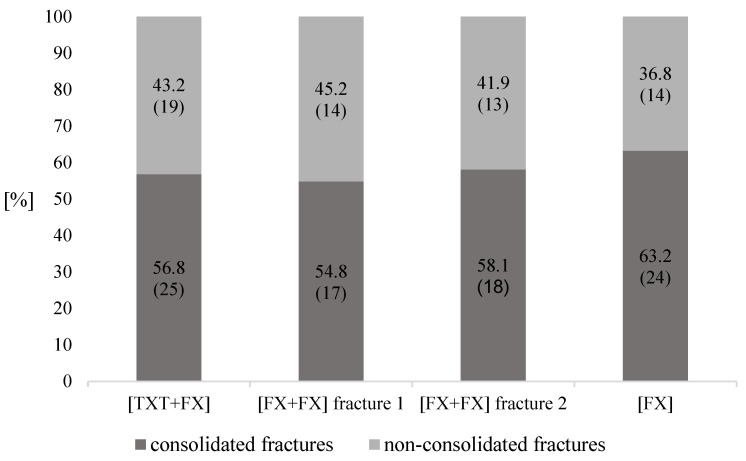
Fracture consolidation and non-consolidation at t_4_. Data are expressed as relative numbers (*n*). Absolut numbers are given in parentheses. Statistical analysis showed no significant difference between the groups regarding fracture consolidation at t_4_ (*p* = 0.84). [TXT+FX]—thoracic trauma and fracture of a long bone, [FX+FX]—fractures of two long bones, [FX]—isolated fracture of a long bone.

The results revealed no statistically significant differences between the three groups regarding RUST at time t_1_ (*p* = 0.09), t_2_ (*p* = 0.07), t_3_ (*p* = 0.28), and t_4_ (*p* = 0.49) (Figure 1). In terms of fracture consolidation (RUST ≥ 10/< 10) between the three groups at t_4_ there was no difference either (*p* = 0.84) (Figure 2). Furthermore, there were no statistically significant differences in consolidation between the groups regarding the anatomical location of non-consolidated fractures (*p* = 0.06) (Table 8 and Table 9).

### 3.6. Thoracic Trauma Scores

In terms of fracture consolidation between TTS_min._ (r = 0.18, *p* = 0.25) and TTS_max._ (r = 0.18, *p* = 0.25) there was no statistically significant correlation found. This was also the case for the parameters AIS_Chest_ and the rate of healed fractures in [TXT+FX] (r = 0.15, *p* = 0.34).

However, there was a significant positive correlation between the PCS and the rate of consolidated fractures in [TXT+FX] (r = 0.33, *p* = 0.03) (Table 10).

## 4. Discussion

Previously published in vivo laboratory data motivated this retrospective clinical investigation into whether accompanying thoracic trauma would negatively influence long bone fracture consolidation. However, the results of this study did not support that hypothesis. The study also investigated whether fractures of two different long bones had an adverse effect on the healing outcome. This hypothesis was also not supported. However, a positive correlation between the PCS and the rate of consolidated fractures was demonstrated.

There are several causes for the discrepancy between the clinical results presented and the influence of a thoracic trauma on fracture healing previously proven in laboratory investigations. One reason might be the suitability of the utilized animal model. The previous reports utilized rodents to conclude the adverse effect theory. In the pre-clinical studies Sprague–Dawley rats [12], Wistar rats [5,9,10,11,13,14] and C57BL/6 mice [15] were used. In some cases, animal models cannot predict the reactions and mechanisms in humans with absolute certainty [26]. Seok et al. showed that this especially applies to inflammatory processes [27]. These inflammatory processes seem to play a decisive role in fracture healing [13,28]. In addition, laboratory animals show a strong genetic similarity to eliminate variations [29], which is not reflected in patient populations. Humans are characterized by a high genetic diversity, which is reflected in the comorbidities of the patients [30].

Another difference between animal and human studies are the test conditions. Pre-clinical studies with rats and mice are standardized, in terms of gender, fixation, defect size and anatomical location [31]. The present clinical retrospective study reflects the clinical reality of population heterogeneity. In addition to trauma mechanisms, patient characteristics and comorbidities, not only fracture location and fracture pattern on the one hand, but also type and extent of the thoracic trauma reflect a part of patients’ heterogeneity. Furthermore, in the pre-clinical work of animal models the fractures are artificially created under standardized conditions [9,10,11,12,13,14,15]. For example, the chest trauma was artificially induced using a pressure wave generator [9,10,11,13,14,15] or a falling weight [12]. This leads to a standardized and homogeneous pattern of thoracic trauma. In contrast to this, the subjects in the present study differed in their trauma mechanisms demonstrating a heterogeneous picture of thoracic injuries. These included osseous, pleural, pulmonary, tracheobronchial, muscular and cardiac lesions. Additionally, a clinical investigation with regard to a single fracture location (e.g., tibia) would be of interest since previous studies have shown that bone consolidation and nonunion development vary depending on anatomical location [32,33]. For example, isolated fractures of the tibia showed higher nonunion rates compared to the humerus [32,33]. These results could be confirmed for all three groups in the present study. The highest number of non-consolidated fractures was found in the lower leg for all three groups, followed by the femur. Besides, it is important to emphasize that the severity of the fractures, the fracture type and the fracture treatment have an impact on fracture healing as well. This fact was not addressed in detail in this study.

Another reason for the discrepancy between the pre-clinical and clinical results is the definition of the time of consolidation. To date, a consistent definition that defines nonunion has not been established [34]. Some authors define nonunion as a fracture that is not healed within at least six months [4,34]. According to other authors and the American Food and Drug Administration (FDA), a nonunion is assumed after a time period of nine months [1,34]. The authors of the clinical studies that examined the influence of TBI on fracture healing have even set the time for the final fracture assessment at twelve months [6,7]. The fracture consolidation in this study was assessed using X-rays six-to-eight months after the definitive operation. Thus, our results can only be compared to a limited extent to the studies, which investigated the influence of a TBI on fracture consolidation. Nonunion rates of 1–10% are reported with individual nonunion rates after open tibial fracture ranging up to 38% [1,32,33]. In our study, the distribution of non-consolidated fractures was 43.2% in [TXT+FX], 45.2 and 41.9% in [FX+FX] and 36.8% in [FX] (Figure 2).

Nevertheless, these comparatively high rates of non-consolidated fractures are in line with some other studies using a RUST cut-off ≥ 10 for healed fractures. In the work of Mehta et al. on bone healing in Gustilo IIIB open tibia fractures, 91% unhealed fractures could be detected in the group that was treated with a fasciocutaneous flap after six months. The group receiving a muscle flap showed a nonunion rate of 67% [35]. In another paper the rate of unhealed tibial fractures was 34% over a mean follow-up period of 26 months if a RUST cut-off ≥ 10 was applied [36]. In contrast, some authors defined bone healing as RUST ≥ 7 [37,38]. This is due to the fact, that a RUST ≥ 7 equates to a minimum of three bridges with cortical callus, at which point a fracture is considered to be radiologically united [39]. Consequently, there are lower rates of non-consolidated fractures in these studies in relation to our study. Nevertheless, the RUST ensures that the assessment of the fractures in the X-ray image, often differing within and between individuals and which leads to varying definitions of unconsolidated fractures, is standardized [34,40]. Thus, the RUST shows excellent intra- and interobserver reliability due to structured X-ray evaluation as well as high correlations with the biomechanical and structural properties of the bone [22,23,37]. Moreover, other authors found that a RUST ≥ 10, as used in this study, correlates more sufficiently with a healed fracture [22,23]. Consequently, further research in this field is required to find the ideal cut-off value for RUST. Furthermore, it is important to emphasize that fracture consolidation could only be evaluated radiologically and not clinically due to the retrospective study design. This is of importance because clinically functional outcomes are also relevant for the evaluation of fracture healing [41]. However, Cekiç et al. found out that the RUST is a reliable tool corresponding directly to the clinical conditions of the patients [36]. In summary, the high rates of unhealed fractures in this study might be mainly caused by the heterogeneity of the study group, the definition of the RUST cut-off and the lacking clinical evaluation.

In addition, the second objective of this study was to assess the predictive value of thoracic trauma scores for fracture consolidation. A positive correlation between one of these scores and reduced fracture healing would have a direct clinical relevance with regard to the risk stratification of delayed fracture healing or nonunion. The statistical results of the present work show that there is a positive correlation between the PCS and the rate of healed fractures in [TXT+FX]. This observation contradicts preclinical experimental data. However, previous literature, elaborating that the AIS_Chest_ does not correlate with complications such as prolonged hospital or ICU stay, extended time of mechanical ventilation, complications and mortality rates [42] is also contrary to the positive correlation of PCS with a higher mortality rate, more frequent ventilatory assistance, lower Horowitz quotient and longer time of hospitalization [43]. The cause for the observed discrepancy cannot sufficiently explained by our study. Reasons responsible for this observation such as potential different treatment modalities remain speculative. Future prospective studies might help to answer this interesting question.

### Limitations

To the best of our knowledge, this is the first clinical study evaluating the influence of a chest trauma on the consolidation of fractures. Nevertheless, several limitations must be pointed out.

Firstly, this was a retrospective, single center study with the common shortcomings of analysis of the prerecorded data [44]. It is further important to emphasize the heterogeneity of our study group. Influencing factors including intensive care unit admission and length of stay as well as surgical treatment strategies were not assessed. Moreover, patients with accompanying injuries including facial, abdominal, soft tissue or spinal injuries without neurological symptoms were not excluded. It has previously been reported that spinal injuries may influence fracture healing [45]. In addition, subjects with a mild TBI were not explicitly excluded from the study, although TBI causes accelerated fracture consolidation and increased callus formation [6,7]. However, this has only been documented for a moderate and severe TBI and has not been demonstrated for mild TBI [6].

Furthermore, it is important to emphasize that the term thoracic trauma encompasses a very heterogeneous field of conditions. Osseous, pleural, pulmonary, tracheobronchial, muscular and cardiac lesions can be included within this term. Subjects with thoracic trauma often need intensive care, endotracheal or non-invasive ventilation, high doses of analgesics or additional surgery. Moreover, they are more likely to develop complications such as pneumonia or acute respiratory distress syndrome [46]. In this work, TTS, PCS and cumulative AIS_Chest_ were used to quantify thoracic trauma. Due to lacking documentation of the Horowitz quotient, we used TTS_min._ and TTS_max._ to determine the real TTS. Regarding the PCS, it is important to emphasize that only pulmonary contusions were included and assessed. In the present study, however, not all patients with thoracic trauma also had a pulmonary contusion. This is a general problem, since thoracic lesions are rarely found in isolation. For example, pulmonary contusions are often associated with flail chest [47]. As a result, the accuracy of both scores is limited.

In addition, the central role of proinflammatory cytokines and immune cells in fracture healing was not considered in this study due to retrospective study design [9,10,13,28]. Consequently, further clinical work with measurement of the cytokine levels (especially IL-1, IL-6, IL-10 and TNF-α) and the release of immune cells in patients with fractures and accompanying chest trauma could contribute to future research resulting in a better understanding of the exact pathologic mechanisms and thus, the therapeutic options.

Finally, it is important to emphasize that the case number of 44 patients in [TXT+FX] could only provide indications of a possible correlation with univariate analysis of thoracic trauma and reduced fracture consolidation. For a more meaningful analysis with a multivariate view and higher reliability as well as validity, a multicenter study with a considerably larger number of cases is required.

## 5. Conclusions

The retrospective analysis did not demonstrate that thoracic trauma results in impaired long bone fracture consolidation of concurrent fractures. The study also did not demonstrate that fractures of two different long bones had an adverse effect on healing outcome of long bone fractures. Due to heterogenicity of the investigated patient population, future and at best registry studies are required to determine the additional influence of treatment and additional clinical variables on fracture healing described in the present study.

## Figures and Tables

**Table 1:** Subjects’ demographic data.

	[TXT+FX]	[FX+FX]	[FX]
patients *n*	44	31	38
age M ± SD (years)	46.1 ± 19.1	48.8 ± 12.7	41.4 ± 20.5
BMI M ± SD (kg/m^2^)	26.5 ± 4.7	27.8 ± 4.6	27.6 ± 7.1
women *n* (percentage)	12 (27.3)	6 (19.4)	14 (36.8)
men *n* (percentage)	32 (72.7)	25 (80.6)	24 (63.2)

Data are expressed as absolute numbers (n), mean (M) and standard deviation (SD). Relative numbers are given in parentheses. There were no statistically significant differences between the three groups regarding age (*p* = 0.22), gender (*p* = 0.28) or BMI (*p* = 0.72). BMI—body mass index; kg—kilogram; m^2^—square meters, [TXT+FX]—thoracic trauma and fracture of a long bone, [FX+FX]—fractures of two long bones, [FX]—isolated fracture of a long bone.

**Table 2:** Subjects‘ trauma scores.

	[TXT+FX]	[FX+FX]	[FX]	*p* Value
ISS M ± SD	24.9 ± 9.1	13.7 ± 3.6	11.9 ± 10.6	<0.001 *
ASA M ± SD	2.5 ± 0.8	2.3 ± 0.7	2.4 ± 0.9	0.50
GCS M ± SD	12 ± 5.1	13.4 ± 3.8	13.4 ± 3.8	0.27

Data are expressed as mean (M) and standard deviation (SD). Significant results are marked with an asterisk (*). ISS injury severity score, ASA American Society of Anesthesiologists classification of physical status, GCS Glasgow coma scale, [TXT+FX]—thoracic trauma and fracture of a long bone, [FX+FX]—fractures of two long bones, [FX]—isolated fracture of a long bone.

**Table 3:** Subjects’ comorbidities.

	[TXT+FX]	[FX+FX]	[FX]
coronary heart disease *n* (%)	3 (6.8)	1 (2.2)	4 (10.5)
cardiac arrhythmia *n* (%)	2 (4.6)	2 (6.5)	2 (5.3)
heart failure *n* (%)	2 (4.6)	2 (6.5)	2 (5.3)
arterial hypertension *n* (%)	11 (25.0)	11 (35.5)	9 (23.7)
peripheral arterial occlusive disease *n* (%)	2 (4.6)	0 (0)	0 (0)
diabetes mellitus *n* (%)	2 (4.6)	2 (6.5)	4 (10.5)
osteoporosis *n* (%)	2 (4.6)	3 (9.7)	0 (0)
hypothyreodism *n* (%)	7 (15,9)	1 (2.2)	3 (7.9)
rheumatic diseases *n* (%)	1 (2.3)	0 (0)	2 (5.3)
kidney failure *n* (%)	0 (0)	0 (0)	1 (2.6)
cerebral insult *n* (%)	1 (2.3)	1 (2.2)	2 (5.3)
depression *n* (%)	4 (9.1)	0 (0)	0 (0)
epilepsy *n* (%)	0 (0)	0 (0)	0 (0)
acohol abuse *n* (%)	4 (9.1)	4 (12.9)	0 (0)
hyperparathyreodism *n* (%)	0 (0)	0 (0)	1 (2.6)
hypoparathyreodism *n* (%)	0 (0)	0 (0)	0 (0)
nikotin abuse *n* (%)	7 (15.9)	7 (22.6)	0 (0)
tumor *n* (%)	1 (2.3)	0 (0)	0 (0)
lung disease *n* (%)	3 (6.8)	0 (0)	3 (7.9)
hyperthyreodism *n* (%)	0 (0)	0 (0)	0 (0)
obesity *n* (%)	3 (6.8)	3 (9.7)	4 (10.5)
HIV infection *n* (%)	2 (4.6)	0 (0)	0 (0)
nervous system disease *n* (%)	1 (2.3)	0 (0)	1 (2.6)
drug abuse *n* (%)	2 (4.6)	1 (2.2)	0 (0)
liver disease *n* (%)	1 (2.3)	1 (2.2)	0 (0)

Data are expressed as absolute numbers (*n*). Relative numbers are given in parentheses. [TXT+FX]—thoracic trauma and fracture of a long bone, [FX+FX]—fractures of two long bones, [FX]—isolated fracture of a long bone.

**Table 4:** Trauma mechanism and length of hospital stay.

	[TXT+FX]	[FX+FX]	[FX]
high-energy trauma/fall from height ≥ 3 m *n* (%)	37 (84.1)	20 (64.5)	23 (60.5)
low-energy trauma/fall from height < 3 m *n* (%)	7 (15.9)	11 (35.5)	15 (39.5)
Length of hospital stay M ± SD (days)	21.6 ± 12.1	16.8 ± 7.9	14.4 ± 11.1

Data are expressed as absolute numbers (*n*). Relative numbers are given in parentheses. [TXT+FX]—thoracic trauma and fracture of a long bone, [FX+FX]—fractures of two long bones, [FX]—isolated fracture of a long bone.

**Table 5:** Fracture single-stage management—definitive treatment.

	[TXT+FX]	[FX+FX] Fracture 1	[FX+FX] Fracture 2	[FX]
plate *n*	19	9	9	13
nail *n*	8	9	4	9
external fixator *n*	0	0	2	0
screws *n*	1	1	1	3
cerclage and k-wire *n*	1	0	0	3
conservative *n*	0	0	2	0
Σ	29	19	18	28

[TXT+FX]—thoracic trauma and fracture of a long bone, [FX+FX]—fractures of two long bones, [FX]—isolated fracture of a long bone.

**Table 6:** Fracture multi-stage management—definitive treatment.

	[TXT+FX]	[FX+FX] Fracture 1	[FX+FX] Fracture 2	[FX]
plate *n*	4	4	9	6
nail *n*	11	7	2	3
external fixator *n*	0	0	0	0
screws *n*	0	1	1	0
cerclage and k-wire *n*	0	0	0	1
conservative *n*	0	0	1	0
Σ	15	12	13	10

[TXT+FX]—thoracic trauma and fracture of a long bone, [FX+FX]—fractures of two long bones, [FX]—isolated fracture of a long bone.

**Table 7:** Distribution of thoracic injuries.

Thoracic Injury	Number of Patients
hemothorax	4
pneumothorax	14
pleural effusion	4
rib fracture (single or multiple)	32
cardiac contusion	1
sternal fracture	4
lung contusion	19
chest contusion	8
lung laceration	4

**Table 8:** Anatomical distribution of non-consolidated fractures at time t_4_.

	[TXT+FX]	[FX+FX]		[FX]
		Fracture 1	Fracture 2	
humerus *n* (percentage)	3 (12.0)	3 (17.7)	0 (0)	2 (8.3)
radius/ulna *n* (percentage)	11 (44.0)	4 (23.5)	7 (38.9)	11 (45.8)
femur *n* (percentage)	6 (24.0)	5 (29.4)	1 (5.6)	5 (20.8)
tibia/fibula *n* (percentage)	5 (20.0)	5 (29.4)	10 (55.5)	6 (25.0)

Data are expressed as absolute numbers (*n*). Relative numbers are given in parentheses. There were no statistically significant differences in consolidation between the groups regarding the anatomical location of non-consolidated fractures (*p* = 0.06). [TXT+FX]—thoracic trauma and fracture of a long bone, [FX+FX]—fractures of two long bones, [FX]—isolated fracture of a long bone.

**Table 9:** Localization of fractures by simplified AO/OTA-classification.

AO	[TXT+FX]	[FX+FX] Fracture 1	[FX+FX] Fracture 2	[FX]
11	3 (6.8)	3 (9.7)	0 (0)	1 (2.6)
12	3 (6.8)	1 (3.2)	0 (0)	2 (5.3)
13	0 (0)	0 (0)	0 (0)	1 (2.6)
21	2 (4.5)	0 (0)	2 (6.5)	4 (10.5)
22	1 (2.3)	3 (9.7)	4 (12.9)	3 (7.9)
23	9 (20.5)	4 (12.9)	5 (16.1)	7 (18.4)
31	0 (0)	2 (6.5)	0 (0)	1 (2.6)
32	9 (20.5)	4 (12.9)	2 (6.5)	8 (21.1)
33	4 (9.1)	4 (12.9)	0 (0)	2 (5.3)
41	5 (11.4)	1 (3.2)	5 (16.1)	5 (13.2)
42	7 (15.9)	6 (19.4)	10 (32.3)	1 (2.6)
43	1 (2.3)	1 (3.2)	1 (3.2)	2 (5.3)
44	0 (0)	2 (6.5)	2 (6.5)	1 (2.6)
Σ	44 (100)	31 (100)	31 (100)	38 (100)

Data are expressed as absolute numbers (*n*). Relative numbers are given in parentheses. [TXT+FX]—thoracic trauma and fracture of a long bone, [FX+FX]—fractures of two long bones, [FX]—isolated fracture of a long bone.

**Table 10:** Thoracic trauma scores.

	[TXT+FX]	*p* Value	Pearson’s Correlation
AIS_Chest_ M ± SD	5.6 ± 4.3	0.34	0.15
PCS M ± SD	2.8 ± 4.4	0.03 *	0.33
TTS_max._ M ± SD	10.4 ± 3.4	0.25	0.18
TTS_min._ M ± SD	5.4 ± 3.4	0.25	0.18

Data are expressed as mean (M) and standard deviation (SD). *p* value und Pearsons’s correlation coefficient are given regarding the correlation between thoracic trauma scores and fracture consolidation. Significant results are marked with an asterisk (*). AIS_Chest_—abbreviated injury scale chest; PCS—pulmonary contusion score; TTS—thoracic trauma severity score, [TXT+FX]—thoracic trauma and fracture of a long bone, [FX+FX]—fractures of two long bones, [FX]—isolated fracture of a long bone.

## Data Availability

The data used to support the findings of this study are included within the article.

## Abbreviations

AIS_Chest_	abbreviated injury scale chest
ANOVA	analysis of variance
ASA	American Society of Anesthesiologists classification of physical status
BMI	body mass index
CT	computed tomography
FDA	U.S. Food and Drug Administration
GCS	glasgow coma scale
ICU	intensive care unit
IRB	institutional review board
ISS	injury severity score
PCS	pulmonary contusion score
RUST	radiographic union scale in tibial fractures
TBI	traumatic brain injury
TTS	thoracic trauma severity score
